# Dual-Purpose Sensing Nanoprobe Based on Carbon Dots from o-Phenylenediamine: pH and Solvent Polarity Measurement

**DOI:** 10.3390/nano12193314

**Published:** 2022-09-23

**Authors:** Anna A. Vedernikova, Mikhail D. Miruschenko, Irina A. Arefina, Anton A. Babaev, Evgeniia A. Stepanidenko, Sergei A. Cherevkov, Igor G. Spiridonov, Denis V. Danilov, Aleksandra V. Koroleva, Evgeniy V. Zhizhin, Elena V. Ushakova

**Affiliations:** 1 International Research and Education Centre for Physics of Nanostructures, ITMO University, 197101 Saint Petersburg, Russia; 2 Research Park, Saint Petersburg State University, 199034 Saint Petersburg, Russia

**Keywords:** carbon dots, protonation, solvatochromism, pH measurement, pH test strip, sensorics

## Abstract

Today, the development of nanomaterials with sensing properties attracts much scientific interest because of the demand for low-cost nontoxic colloidal nanoprobes with high sensitivity and selectivity for various biomedical and environment-related applications. Carbon dots (CDs) are promising candidates for these applications as they demonstrate unique optical properties with intense emissions, biocompatibility, and ease of fabrication. Herein, we developed synthesis protocols to obtain CDs based on o-phenylenediamine with a variety of optical responses depending on additional precursors and changes in the reaction media. The obtained CDs are N-doped (N,S-doped in case of thiourea addition) less than 10 nm spherical particles with emissions observed in the 300–600 nm spectral region depending on their chemical composition. These CDs may act simultaneously as absorptive/fluorescent sensing probes for solvent polarity with $∆S/∆E_{N}^{T}\;$ up to 85, for $∆E_{N}^{T}$ from 0.099 to 1.0 and for pH values in the range of 3.0–8.0, thus opening an opportunity to check the pH in non-pure water or a mixture of solvents. Moreover, CDs preserve their optical properties when embedded in cellulose strips that can be used as sensing probes for fast and easy pH checks. We believe that the resulting dual-purpose sensing nano probes based on CDs will have high demand in various sensing applications.

## 1. Introduction

In the past years impressive progress in sensing technologies has been achieved due to the development of spectroscopic and microelectronic devices along with success in synthetic methods including organic and/or inorganic materials and nano composites [1]. This has led to the development of many technologies, including more precise touch sensors [2], fast devices for movement detection, and chemical sensors for media monitoring. Now, the field of chemical sensing is undergoing tremendous growth in areas from environmental control [3], healthcare [4] and the food industry [5] to materials examination such as metal corrosion [6] and oil quality. Among sensing methods, optical techniques have attracted attention because of the high potential in in vivo imaging and monitoring at the nanoscale. In optical response engineering, these probes are expected to demonstrate not only changes in absorption/reflectance but also changes in their fluorescent parameters, e.g., photoluminescence intensity, peak position, excited-state relaxation, etc. [7]. In this view, colloidal nanoparticles provide key advantages such as increased emission quantum yield, control of optical transitions in a wide spectral range, excited-state engineering via plasmon-enhanced fluorescence, and resonant energy transfer, to name a few.

Considering biomedical applications and the food industry, sensing probes should also be biocompatible and eco-friendly, demonstrate a stable response under changing media and intense/continuous light exposure, and allow incorporation in various matrices for solid-state sensors. All of the above-mentioned criteria are met by carbon nanoparticles, making them a highly attractive subject of research. Due to their ability to support several optical centers, their excitation-dependent emission facilitates the sensitivity of chemical sensing via distinguishable spectral changes for different optical centers including ratiometric responses. The possibility to functionalize the surface of carbon nanoparticles allows a further increase in sensitivity to specific analytes such as nitrite and ascorbic acid [8] and enzymes [9], and expansion to their range of their applications. Carbon dots (CDs) have been already used for detection of solvent polarity [10,11,12], e.g., water content in polar solvents [10,12,13,14,15], pH values [9,16,17,18,19,20,21], temperature and specific analytes such as proteins or metal ions. Commonly, pH sensing is based on labeling by certified agents with known optical response. However, this approach requires a complicated multistep preparation procedure of the complexes to examine the media. Thus, CDs that support several optical centers with different chemical composition may act as a novel sensing probe. Considering in vivo imaging and measurement of the media parameters in biological objects, CDs with optical transitions in the red and near-infrared spectral regions are in demand because they provide high signal-to-noise ratio and the possibility to shift excitation wavelengths to the visible spectral range to avoid autoluminescence from tissues [22]. From this point of view, CDs synthesized from o-phenylenediamineo (o-PD), a common and nontoxic organic precursor [23], with emission in the green to red spectral range are highly attractive for bio-imaging and related applications due to improvements in the signal-to-noise ratio. The CDs from o-PD have been applied to detect metal ions such as Cu^2+^ that have been found to affect the activity of coenzymes which influence Alzheimer’s disease when present in cells [24]. In addition, these types of CDs can be used to detect pH, an important parameter in diverse physiological processes [25,26]. M. Zhang et al. showed that o-PD based CDs exhibit a linear response of PL intensity to a change in pH from 1.0 to 13.0 with reversible emission [27]. CDs synthesized from a mixture of o-PD, p-PD, and dopamine demonstrate a pH response to the PL intensity under two-photon excitation, as was recently shown in Ref. [28]. Furthermore, CDs based on o-PD also showed a change in the position of the PL band position for a set of solvents from tetrahydrofuran to water [29] and for the mixture of water and dioxane [30]. However, most of the developed probes are designed for pH or solvent polarity sensing only, which limits their further utilization in sensorics of objects with non-pure solvents, for example, the mixture of ethanol and water, which is very important for living tissues [7].

Herein, we developed synthesis protocols to obtain CDs based on o-PD that demonstrate a distinguishable response in absorption and photoluminescence to both solvent polarity and pH. It was achieved by a change of the synthesis parameters leading to a variety of chemical compositions and optical responses of nanoparticles. Based on these CDs, filter paper/cellulose test strips that can be used as a simple disposable sensing probe for colorimetric pH determination with a smartphone were fabricated.

## 2. Materials and Methods

### 2.1. Materials

O-phenylenediamine (flaked, 99.5%), hydrochloric acid (38%), benzoic acid (ACS reagent, ≥99.5%), and thiourea (>99%) were purchased from Sigma Aldrich. Rhodamine 6G was purchased from Sigma-Aldrich (Darmstadt, Germany). Toluene (>99.5%), chloroform (>99.8%), acetone (>99.8%), acetonitrile (>99.8%), and isopropanol (>99.8%) were purchased from Ekos-1 (Moscow, Russia). Deionized water (Milli-Q water) and ethanol (>96%, Vekton, Saint Petersburg, Russia) were used as solvents. All chemicals were used as received.

### 2.2. Carbon Dot (CD) Synthesis

CDs samples were obtained by solvothermal heating of the precursors solution. CD-w was synthesized from 0.5 g of o-PD in 25 mL of distilled water. CD-HCl was synthesized similarly to CD-1 with additions of HCl to form acid media (pH = 2). CD-BA-w was synthesized from 0.5 g of o-PD and 0.56 g of benzoic acid dissolved in 25 mL of water. CD-TU-w was synthesized from 0.5 g of o-PD and 0.7 g of thiourea dissolved in 25 mL of water. The amounts of precursors including oxygen for all samples is summarized in Table S1 (SI). All precursor mixtures were heated at 180 °C in Teflon-lined stainless-steel autoclaves for 6 h. After the reaction, the autoclave was naturally cooled to room temperature. Colloidal solutions were purified from large particles and agglomerates using a filter with a 0.22 μm membrane. To remove unreacted precursors and molecular fluorophores, the filtered solutions were transferred to a dialysis tube with a molecular weight of 1000–2000 Da for dialysis for 24 h. The concentrations of the prepared CDs stock solutions were 10–40 mg/mL. For optical measurements, stock solutions were diluted to obtain clear solutions with optical density of 0.1 at the wavelength of interest.

### 2.3. Experimental Setup

Transmission electron microscopy (TEM) images were obtained on a Libra 200FE (Zeiss, Oberkochen, Germany). Atomic force microscopy (AFM) measurements were carried out using a Solver PRO-M microscope (NT-MDT, Moscow, Russia) in the semi-contact mode. For AFM measurements, 10 μL of light-brown CD solutions were spin-casted onto the mica substrates at 2000 rpm for 30 s. XPS measurements were performed on an Escalab 250Xi photoelectron spectrometer with AlKα radiation (photon energy 1486.6 eV). Measurements were performed in the constant pass energy mode at 100 eV for the survey XPS spectrum and at 50 eV for the core level spectra of single elements, using an XPS spot size of 650 μm. Raman spectra were measured on an inVia (Renishaw, Wotton-under-Edge, UK) microspectrometer equipped with a 20× object and a 514 nm laser source. Fourier-transform infrared (FTIR) spectra were recorded on a Tenzor II infrared spectrophotometer (Bruker, Billerica, MA, U.S.) in an attenuated total reflection mode. Absorption spectra were measured on a spectrophotometer UV-3600 (Shimadzu, Kyoto, Japan). PL spectra and PL excitation-emission (PLE-PL) maps in the UV-Vis range were collected on a Cary Eclipse (Agilent, Santa Clara, CA, USA). Time-resolved PL measurements were performed on a confocal microscope MicroTime 100 (PicoQuant, Berlin, Germany) equipped with a 3× objective (NA = 0.1) and a 405 nm pulsed diode laser. PL decay curves were fitted by a biexponential function: $I\left(t\right)=I_{0}+A_{1}e^{-t/\tau_{1}}+A_{2}e^{-t/\tau_{2}}$. The average PL lifetime has been calculated as $\langle \tau \rangle =\sum A_{i}\tau_{i}^{2}/\sum A_{i}\tau_{i}$. pH was measured with a pH meter HANNA HI 2211 (HANNA Instruments, Woonsocket, RI, USA).

## 3. Results and Discussion

### 3.1. Chemical Reactions during CDs Synthesis

During heating of the precursors mixture in an autoclave, o-PD may undergo an oxidation, the speed and yield of which depend on the amount of oxygen, pH of the solution, and influence of additional precursors. Figure S1 shows the plausible chemical reactions that lead to the formation of carbonized N-doped domains within the CDs. At the first stage, the oxidation of o-PD in a neutral environment may result in the formation of 2,3-diaminophenazine (2.3-DAPN), as was recently shown in Ref. [31] (Figure S1a). Next, oxidation and dehydration can result in the formation of both cross-linked (Figure S1b) and linearly linked 2,3-DAPN molecules (Figure S1c), which act as building blocks for N-doped CDs. In this process, oxygen plays the key role, which is necessary for the initial formation of an imine derivative, as well as the pH environment for subsequent carbonization of the domain. For all CDs synthesis, the ratio of o-PD molecules to oxygen atoms was estimated as 8.6 to 1 (details are provided in Table S1). An increase in the amount of oxygen leads to a faster course of the reaction and to an increase in the domain carbonization, but also to tarring of peripheral molecular groups that allow CDs to be soluble in water and prevent their aggregation. Thus, the selection and control of the oxygen to precursors ratio are important for ensuring a reproducible mass output and optical properties of the resulting CDs. While the oxygen amount can be varied by increasing the free volume of the autoclave or the addition of different oxidizing agents, the pH can be changed in more controlled way by the addition of acid/base to the precursor mixture. With a decrease in pH the following processes may occur: (i) in an acidic environment, the oxidation rate increases due to a significant increase in the electrochemical potential of oxygen according to the Nernst equation (E^0^ = +1.2291 V) [32]; (ii) the mesomeric structure of o-chinondiimine with the predominant charge localization in the third position is stabilized by additional protons and by the subsequent attack of o-PD on it, which leads to an increase in the reaction speed [33]; (iii) mainly one of the amino groups of the o-PD is protonated, while the basic and nucleophilicity of the second amino group is greatly reduced due to the conjugation and redistribution of the electronic pair, which leads to a decrease in its reaction rate with o-chinondiimine. We have chosen HCl (CD-HCl) and benzoic acid (CD-BA) for checking these assumptions. The benzoic acid itself has an electron deficient benzene ring while it does not enter the reaction of nucleophilic substitution into the ring and does not participate in the formation of a conjugated carbon domain in CDs. The main reactions may pass through the carboxyl group with the formation of amides at the CD’s surface, which leads to their hydrophobization and a change in morphology between layers due to sterical exposure. Thus, the plausible structure of CD-BA may be less dense with a smaller number of conjugated and aromatic domains within the CD. These changes in morphology will be considered later in the text.

Another way to engineer structural and optical properties of CDs is a heteroatom doping by using additional N,S-containing precursors. For this, we have chosen thiourea (CD-TU sample) which allows to form N,S-doped CDs. In an aqueous solution, thiourea is hydrolyzed with the formation of hydrogen sulfide, which promotes a formation of 2,3-diaminophenotiazin and its subsequent carbonization. Direct reaction between o-PD and thiourea is also possible by the scheme shown in Figure S2. These assumptions are discussed later in the text in comparison with experimental observations of structural and optical parameters.

### 3.2. Morphology and Optical Centers

**Figure 1:**
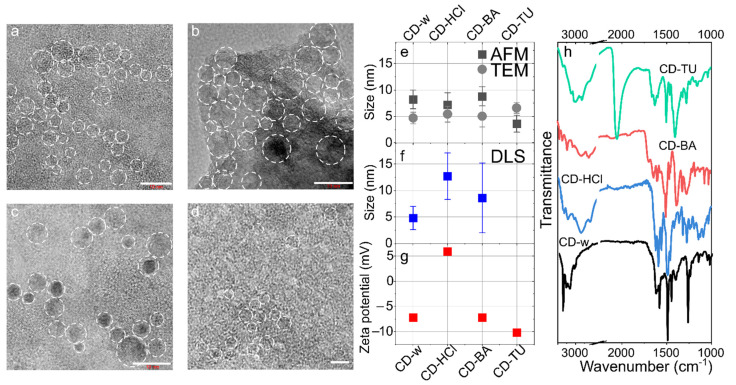
(**a**–**d**) Transmission electron microscopy (TEM) images of CDs: CD-w (**a**), CD-HCl (**b**), CD-BA (**c**), CD-TU (**d**). CDs are highlighted by dashed circles. The size bar is 10 nm. CDs sizes estimated from AFM (black squares) and TEM (grey circles) images (**e**) and DLS measurements (**f**). (**g**) ζ-potential of CDs. (**h**) FTIR spectra of CDs: CD-w (black), CD-HCl (blue), CD-BA (red), CD-TU (green).

The TEM images (Figure 1a–d) show that CDs are spherical particles with a size not exceeding 10 nm. CDs synthesized with acid addition (CD-HCl and CD-BA) exhibited more carbonized structures, as is seen from the presence of the crystal fringes in the TEM images (Figure 1b,c). The average sizes estimated from TEM analysis were 4.7 ± 1.1, 5.4 ± 1.5, 5.0 ± 2.0, and 6.6 ± 1.0 nm for CD-w, CD-HCl, CD-BA, and CD-TU, respectively (Figure S3). The CDs heights from the AFM images (Figure S4a–d) were also less than 10 nm and were very similar to those estimated from the TEM images (Figure 1e). However, the sizes estimated from the DLS spectra were 4.8 ± 2.2, 12.8 ± 4.4, and 8.6 ± 6.4 nm for CD-w, CD-HCl, and CD-BA, respectively. For the CD-TU sample, CDs were aggregating at a neutral pH, resulting in observed sizes of ~100 nm. It can be inferred that the size of nanoparticles is almost independent of the types of additives during CDs synthesis based on o-PD, whereas the chemical composition affects the stability of the colloidal solution of CDs, and for CD-TU, results in CD aggregation. The ζ-potential was slightly negative with values not exceeding −11 mV and was −7.2 mV for CD-w and CD-BA and −10.2 mV for CD-TU. At the same time, for the CD-HCl sample, the ζ-potential was +5.9 mV, which indicates that the amine groups are protonated when CDs are synthesized in an acidic environment.

In Figure S5a, the full survey XPS spectra show that CDs mostly consisted of carbon and nitrogen with low amounts of oxygen (and sulfur for CD-TU) with a ratio of nitrogen to carbon atoms of 18–21% (Figure S5b), which agrees well with the N-doped structure predicted in Section 3.1 (Figures S1 and S2). The high-resolution spectrum of C 1 s shows that the band consists of two peaks—strong at 285.0 and weak at 288.7 eV—corresponding to C–C and O=C–N, respectively (Figure S5c). The N 1s band is rather broad and shifted from 399.2 eV for CD-w to 399.4 eV for CD-HCl, CD-BA and CD-TU, indicating the presence of imine and amine bonds with an increase in the latter bonds for synthesis with additives (Figure S5d). For CD-TU, S 2p is observed at 162.9 eV with a shoulder at 164 eV corresponding to C–S bonds.

The Raman spectra shown in Figure S6 are typical for CDs based on o-PD: the most intense peak at 1360 cm^−1^ is attributed to breathing vibrations of benzene rings; the peaks at 1255 and 1380 cm^−1^ correspond to C-NH bending and C-N stretching [33], respectively; the peaks at 1402 and 1487 cm^−1^ are attributed to phenazine-like structures and 2,3-DAPN [34], respectively. It is seen that these peaks are shifted for CD-HCl, CD-BA, and CD-TU compared with the CD-w sample by 5–8 cm^−1^, which may indicate a shorter C-N or C=N bond length. Furthermore, the peaks attributed to phenazine-like structures are more pronounced in CD-HCl, CD-BA, and CD-TU samples, thus indicating the formation of a larger amount of phenazine derivatives, as was predicted from chemical reactions in Section 3.1 (Figures S1 and S2). The FTIR spectrum of CD-w shown in Figure 1h as a black line demonstrates intense and narrow peaks at 3385, 3365, 1630, and 1277 cm^−1^, which can be attributed to symmetric, asymmetric vibrations, scissoring of -NH_2_, and C-N stretching, respectively. Peaks at 3000–3070 and 1540–1600 cm^−1^ can be attributed to stretching vibrations of C-H and C=C (ring), respectively. Thus, CD-w consists of N-doped aromatic domains with a surface rich in amine groups. For the CD-HCl sample, a peak at 2840 cm^−1^ with additional peaks at 2500–2600 cm^−1^ can be attributed to structures similar to imidazole or protonated amines, which agrees well with measurements of the CDs surface charge. These peaks are not intense but are also observed in spectra for both CD-BA and CD-TU. For CD-BA, in addition to peaks observed for CD-HCl, a peak at 1700 cm^−1^ emerges, which can be attributed to C=O/N-C groups in amides. For CD-TU, a strong peak at 2048 cm^−1^ typical for N=C=S is observed. Thus, the addition of acids leads to formation of an N-doped phenazine-like carbon network and to larger degree of protonation of amino groups at the CDs surface, whereas addition of thiourea leads to a formation of N, S-codoped aromatic carbon domains within CDs. These observations agree well with the predicted chemical structure of CDs demonstrated in Figures S1 and S2.

### 3.3. Optical Properties Depending on CDs Morphology

**Figure 2:**
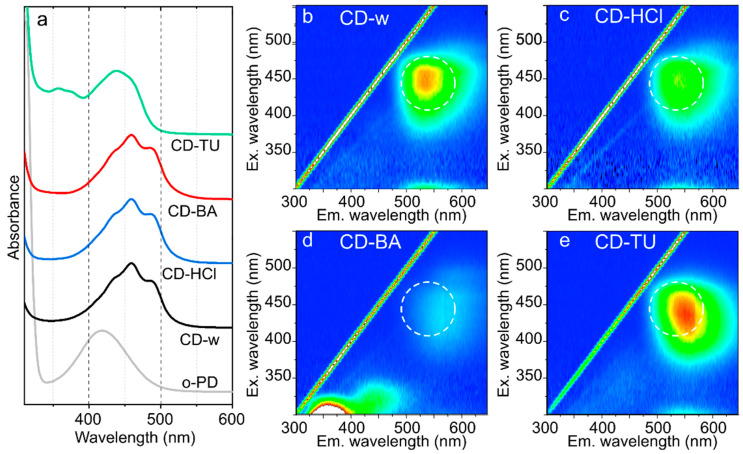
(**a**) Absorption spectra of o-PD (grey) and CDs: CD-w (black), CD-HCl (blue), CD-BA (red), CD-TU (green). (**b**–**e**) PL excitation-emission maps of CD-w (**b**), CD-HCl (**c**), CD-BA (**d**), CD-TU (**e**). For comparison, the PL band of CD-w is shown as a dashed circle.

In the absorption spectra shown in Figure 2a, the formation of carbon domains of linked 2,3-DAPN is observed by a red shift of the main absorption band from 420 nm for pristine o-PD in aqueous solution to 460 nm for CD-w, CD-HCl, and CD-BA, which may indicate the formation of larger carbon domains together with its doping with heteroatoms [31]. Along with the red shift, the main absorption band exhibits a complex structure and can be deconvoluted to three peaks at 435, 460, and 486 nm. For CD-TU, the main absorption peak is red shifted by only 20 nm compared with that of o-PD but with appearance of peaks at 350–380 nm which can be attributed to a C=S absorption [35].

The PL excitation-emission maps are shown in Figure 2b–e. For CD-w, the PL band is centered at 535 nm with maximal excitation at 445 and ~270 nm which correspond to peaks in the absorption spectrum (Figure 2b). The increase in acidity by HCl does not result in any noticeable changes (Figure 2c), whereas the addition of benzoic acid results in appearance of intense blue emission with a PL peak at 360 nm while the main PL band is red shifted to 570 nm (Figure 2d). For CD-TU, the main PL band is centered at 550 nm with maximal excitation at 435 and ~270 nm, whereas absorption peaks at 350–380 nm are not involved in emission as they are not seen in the PL excitation-emission map (Figure 2e). The optical parameters of all CD samples are summarized in Table S2. From these observations it can be assumed that for all o-PD CDs, the absorption peaks at 260–280 and 435–440 nm ranges contribute to the emission band centered at 535–570 nm. S-doping results in a slight shift of the emission band by only 15 nm. The most interesting changes are observed for CD synthesized with benzoic acid: there formed intense blue-emissive centers within CDs, which can be attributed to the incorporation of the benzoic acid molecules within carbon domains, leading to a loosening of the structure and formation of smaller sp^2^-domains. It is worth noting that for the CD-w sample, the cooling and heating of the solution slightly affected the optical properties of the CD-w solution (Figure S7a and Table S3), resulting in PL peak shift within 10 nm. Additionally, the optical properties remain the same when the CD-w sample was stored under ambient atmosphere at 4–8 °C (Figure S7b and Table S3). Thus, these objects are attractive for sensing applications under different conditions.

### 3.4. Solvent Polarity Probe

The presence of both -NH and -CH groups at the surface of CDs provides a possibility to dissolve them in media with different polarity. The optical properties of CD-w and CD-TU were monitored while dispersed in: toluene (0.099), chloroform (0.259), acetone (0.355), acetonitrile (0.46), isopropanol (0.546), ethanol (0.654), and water (1.0). The normalized polarity values ($E_{N}^{T}$) are shown in brackets.

**Figure 3:**
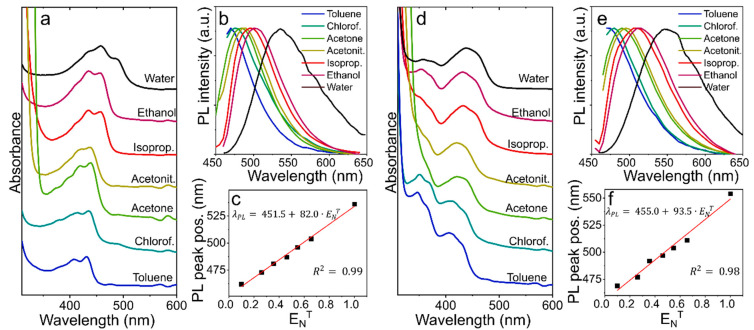
Dependence of optical properties on solvent polarity for CD-w (**a**–**c**) and CD-TU (**d**–**f**). (**a**,**d**) Absorption spectra. (**b**,**e**) Normalized PL spectra excited at 450 nm. (**c**,**f**) PL peak position (excited at 450 nm) depending on normalized solvent polarity ($E_{N}^{T}$) of the solvent. The linear fit is shown by straight red lines. See the main text for details.

For CD-w, the change of the solvent from water to alcohols (ethanol or isopropanol) resulted in the absorption peak at 485 nm disappearing, whereas decreasing the polarity of the solvent starting from isopropanol to toluene led to a blue shift of the main absorption band from 460 to 430 nm (Figure 3a). The PL excitation-emission maps for CD-w (Figure S8) show that in non-polar solvents, both excitation and PL peak were blue shifted together with emergence of vibrational structures in the PL spectra. These observations are similar to those for organic dyes, for example dapoxyl sulfonyl ethylenediamine [36] and also CDs synthesized from para-phenylenediamine (p-PD) [11]. The PL band excited at 450 nm underwent similar changes with a more pronounced shift between CD-w in aqueous and ethanol solution (Figure 3b). The PL peak position (excited at 450 nm) depends linearly on $E_{N}^{T}\;$, as shown in Figure 3c, and it is blue shifted by 73 nm (∆S). The dependence of the peak position (excited at 450 nm) on $E_{N}^{T}$ can be fitted by linear function: $\lambda_{\mathrm{PL}}=451.5+82.0\cdot E_{N}^{T}\;\left(R^{2}=0.99\right)$.

For CD-TU, the main absorption band was also blue shifted from 440 to 415 nm when the polarity of the solvent was decreased. It should be noted that absorption in the 350–380 nm spectral region increased with a decrease in $E_{N}^{T}$ (Figure 3d). In the PL excitation-emission maps for CD-TU (Figure S9), it is seen that the efficient excitation and emission were blue shifted with decreasing the polarity of the solvent, which was accompanied by an increase in the PL intensity in nonpolar or less polar solvents (toluene, chloroform, and acetone) compared with alcohols. This observation suggests that in more polar solvents, additional losses of energy occur, including solvent relaxation [36]. Since the PL band of CD-TU in water was observed at 550 nm under 450 nm excitation, the PL peak shift was more pronounced ($∆S/∆E_{N}^{T}\;$≈ 85 nm) with PL peak position dependent on solvent polarity as: $\lambda_{\mathrm{PL}}=455.0+93.5\cdot E_{N}^{T}$ $\left(R^{2}=0.98\right)$.

These observations suggest that the optical centers formed in CDs in their excited state have greater polarity than in their ground state, which was also observed for aromatic molecules [37] and o-PD based CDs [30]. In highly polar solutions such as water ($E_{N}^{T}$ = 1), the excited state stabilized through solvent relaxation resulting in energy loss and narrowing of the energy bandgap (redshift of PL band) [37]. The change in measured PL QY values for CD-w in alcohols and water confirms the interaction of solvent dipoles with the CD’s excited state (Table S4). With an increase in the solvent polarity, PL QY decreases [29]. It is worth noting that distinguishable positive solvatochromism of the PL band can also be detected under UV light (365 nm), which corresponds to a widely spread and easily accessible UV LED chip. The dependencies of the PL spectra excited at 360 nm are shown in Figure S10. The distinguishable color change of the solutions in different solvents under 265 nm and 365 nm excitation shown in Figure S11 suggests an opportunity to check the solvent polarity using a digital camera on a smartphone [11].

### 3.5. pH Probe

**Figure 4:**
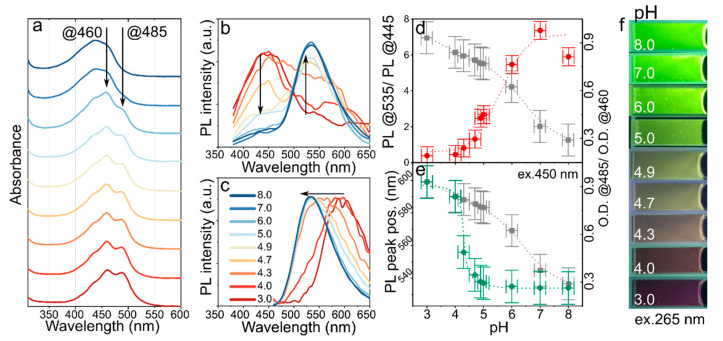
Optical properties of CD-w dissolved in water at different pH. (**a**) Absorption spectra; arrows are used as a guide for eye, (**b**) PL spectra excited at 360 nm; arrows show change in the PL intensity. (**c**) PL spectra excited at 450 nm. Color of lines in (**a**–**c**) is given in the legend of (**c**). (**d**) Ratio of PL intensities at wavelengths 535 and 445 nm in PL spectrum excited at 360 nm (red diamonds) versus pH value of solution. (**e**) PL peak position under 450 nm excitation (green circles) versus pH value of solution. In (**d**,**e**), the ratio of optical density at 485 nm to 460 nm is shown by grey squares. (**f**) Photo of CD-w aqueous solutions with different pH under 265 nm excitation.

pH sensing in biological objects including cells and tissues is a current emerging area of applications for colloidal nanoparticles [17]. In this case, CDs are the most attractive probes because of their nontoxicity and ease of functionalization. The estimation of pH can be achieved by absorptive and fluorescent methods, including ratiometric methods where intensity of two peaks in the emission band are compared. We examined the possibility of CD-w detection of pH. Aqueous solutions with varied pH were prepared, to which a constant amount of CD-w was added. The absorption and emission of CD-w demonstrated a clear dependence on pH in aqueous solution in the range from 3.0 to 8.0 (Figure 4). The vibrational structure of the main absorption band disappeared when the pH increased to 8.0, whereas decreasing the pH to 3.0 resulted in redistribution of optical density in the main absorption band and wavelengths 485 and 460 nm with almost constant optical density observed at 435 nm (Figure 4a). The ratio of optical densities observed at 485 and 460 nm (O.D.@485/O.D.@460) versus pH value is shown by grey squares in Figure 4d,e. This data can be approximated by linear function both for the whole pH range and for acidic/base environment as shown in Figure S12.

The already developed pH sensing probes based on CDs implement change in the PL intensity during the protonation/deprotonation process [38]. It was shown that using several excitation sources and the development of nanoparticles with a ratiometric response to pH [18], improves the sensitivity and expands the pH detection range. It is worth noting that the pH probes supporting ratiometric measurements for both absorption and emission are attractive because the ratio of the emission intensities at two excitation wavelengths is less dependent on such parameters as sensitivity of the detector, fluctuations in the light source intensity or concentration of particles [37]. Moreover, it was recently shown in Ref. [31] that the amines on the surface of CDs based on o-PD can be efficiently protonized/deprotonized by changing the pH of the medium, resulting in a shift of the PL peak from the yellow to red spectral region when increasing the pH from 1 to 7.

The PL excitation-emission maps shown in Figure S13 demonstrate that in a more acidic environment (pH up to 4.3), an emission at 300–450 with effective excitation at 300–400 emerged, accompanied by a redshift of the main PL band to ~600 nm (pH = 3.0) (Figure S13a–d). With an increase in pH from 4.7 to 8.0, the blue emission disappeared and the main PL band was shifted to 535 nm, which could be efficiently excited at 250–310 nm and 375–500 nm (Figure S13e–i). When comparing the PL spectra of CD-w excited at 360 nm shown in Figure 4b, it is seen that the change in pH resulted in changes in the intensities of PL peaks at 445 and 535 nm. This intensity redistribution is similar to that observed for ratiometric pH probes. These probes have been shown to be more promising, as the intensity of PL can be efficiently quenched with a change in the chemical environment in the vicinity of CDs [17,18,19], enabling a sensitivity of up to 0.067 pH units [16]. The changes in the intensity ratios at 445 and 535 nm (PL@535/ PL@445) are plotted in Figure 4d. It is seen that at the ends of the pH range, e.g., at pH < 4.0 and pH > 7.0, the ratio saturates; in the pH range from 4.3 to 6.0 the intensities ratio can be fitted by a linear function: ${\mathrm{PL}}_{535}/{\mathrm{PL}}_{445}=-10.12+2.53\cdot \mathrm{pH}$, and pH can be estimated as follows: $\mathrm{pH}=4.01+0.39\cdot \left({\mathrm{PL}}_{535}/{\mathrm{PL}}_{445}\right)$ with $\left(R^{2}=0.98\right)$. The observed redistribution of the PL intensity excited at 350 nm could indicate an interaction between the energy levels existing in CDs via charge/energy transfer suppression or improvement. At the same time, the PL band excited at 450 nm was blue shifted with increasing pH, as shown in Figure 4c. This suggests the opportunity to estimate pH value by a PL peak position excited in the spectral range 375–500 nm. The PL peak position versus pH value also exhibited a nonlinear dependence with a sharp PL peak position change in the pH range 4.0–5.0, allowing sensitive detection of pH in this region. The observed shift is typical for organic dyes and is caused by protonation/deprotonations of the groups resulting in a decrease/increase in the bandgap [37]. This dependence can be fitted by a logistic function with fixed minimum and maximum of the PL peak position value at 535 and 600 nm, respectively: ${\mathrm{PL}}_{\mathrm{peak}}=535+\left(600-535\right)/(1+\left(\mathrm{pH}/4.2{)}^{22}\right).$ Since the main PL band can be excited also in the deep UV region, the emission color change with pH can be examined under 265 nm UV lamp excitation, as shown in Figure 4f. It is clearly seen that the emission color changes from dark wine to bright green with pH changes from 3.0 to 8.0.

Thus, CD-w sample can be used as pH probe supporting three different methods for pH estimation. This helps expand the pH range of interest and available experimental methods. pH values were calculated from absorption (${\mathrm{pH}}_{\mathrm{abs}}=9.98-0.60\cdot \mathrm{O.D}._{485}/\mathrm{O.D}._{460}$) and emission excited at 360 nm (${\mathrm{pH}}_{r}=4.01+0.39\cdot {\;\mathrm{PL}}_{535}/{\mathrm{PL}}_{445}$), and can be also estimated from PL peak position excited at 450 nm. It should be noted that in a recent review, the pH estimation by fluorescent methods using two different excitation wavelengths was highlighted as an attractive opportunity to improve existing measurement methods [7].

### 3.6. Dual-Purpose Sensing Probe and Sensing Test Strip

**Figure 5:**
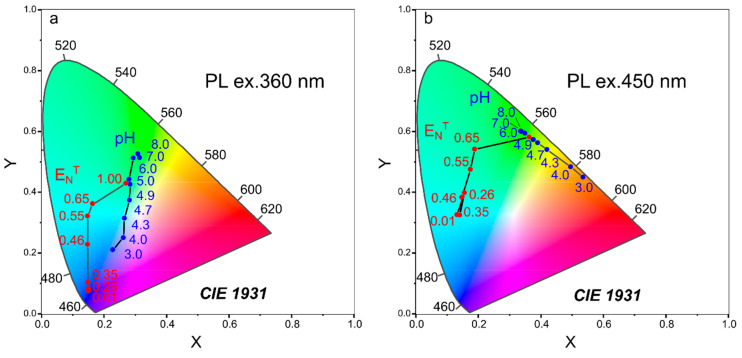
CIE diagram representing the changes in the emission spectrum of CD-w with changing solvent polarity ($E_{N}^{T}$, red circles) and pH (blue circles) of CD aqueous solutions. Excitation wavelengths: 360 nm (**a**) and 450 nm (**b**).

To understand if CDs developed in this work are suitable for simultaneous measurement of pH and solvent polarity, the obtained data on emission changes with media parameters was plotted in a CIE (Commission Internationale de Photométrie) graph shown in Figure 5. Indeed, under changing solvent polarity and pH, the emission parameter changes are distinguishable for both excitation wavelengths, 360 nm (Figure 5a) and 450 nm (Figure 5b). At the same time, the comparison of Figure 5a,b suggests that when excited at 450 nm, the dependencies of the emission color on solvent polarity $(E_{N}^{T})\;$ or pH are more pronounced.

One of the main challenges in sensorics, especially for bio-objects, is to detect pH in a media containing non-pure water or in water mixed with other solvents such as alcohol. Firstly, we checked the optical properties of CD-w solutions with added salts of Ca, Co, Cu, and Fe. From the absorption spectra (Figure S14) it can be observed that the presence of Fe^3+^ affected the position of the band and the peak intensity, whereas the presence of Ca^2+^, Co^2+^ or Cu^2+^ led to a decrease in the intensity of the whole band with the maintenance of the O.D.@485/O.D.@460 ratio. The PL band with added Co^2+^ and Cu^2+^ maintained the position of the maximum along with a decrease in intensity with increasing concentration of ions (Figure S14d,f). The addition of Ca^2+^ ions resulted in a red redshift shift of the PL maximum from 535 to 585 nm and a decrease in its intensity (Figure S14b), which could interfere with the pH estimation. The addition of Fe^3+^ ions led to a dramatic change in the peak position and intensity of CDs, as shown in Figure S14h. A similar dramatic sensitivity to Fe^3+^ ions has been attributed to the possible electron transfer on optical centers and a strong binding affinity to CDs surface groups [39]. These results suggest that CD-w may be utilized as sensor even for non-pure water containing such ions as Ca^2+^, Co^2+^, Cu^2+^. To further check the possibility of the pH and solvent polarity measurements, the optical signals from CD-w dissolved in a mixture of ethanol and water (1/1) with a varied pH of 4.1 (sample 1), 4.7 (sample 2), and 5.9 (sample 3) were examined. The absorption and PL spectra excited at 360 and 450 nm are shown in Figure S15. From Figure S15a it can be seen that the main absorption band underwent a slight blues-shift accompanied by a change in the optical density ratio monitored at 485 and 460 nm, indicating the change in pH in the sample set. The PL band excited at 350 nm contained two main peaks at ~515 and ~440 nm with a varying intensity ratio of the peaks (Figure S15b). Such a blue shift of the main peaks indicates the decreased polarity of the media, whereas the varying intensity ratio reflects the pH change. The PL band excited at 450 nm also contained two peaks, with the maximum PL intensity observed at ~565, ~520, and ~515 nm for samples 1, 2, and 3, respectively (Figure S15c). The PL peak observed at 565 nm reflects the decreased pH of the media. To estimate the amount of ethanol, peak positions of the absorption band and PL peak positions were used to calculate $E_{N}^{T}$ and were plotted on the linear dependencies of the optical parameters on $E_{N}^{T}$ (Figure S16a). Both the absorption and PL peak indicate that the solvent used was the mixture of ethanol and water. The $E_{N}^{T}$ values were estimated from linear dependencies of the peak position on polarity and summarized in Table 1. From the $E_{N}^{T}$_PL_ values, the amount of ethanol in the water can be predicted as 63 and 79% for samples 2 and 3, respectively. For sample 1, since the PL excited at 450 nm was strongly red shifted, the amount of ethanol was estimated by absorption peak position (94%), which shows poor relation to the real value. The pH was estimated by the ratiometric method based on changes in both absorption (${\mathrm{pH}}_{\mathrm{abs}})\;$ and the PL intensity ratio excited at 360 nm (${\mathrm{pH}}_{r})$ shown in Figure S16b and summarized in Table 1. The calculated pH follows the trend of measured values, and for slightly acidic media shows only 10% errors. It should be noted that pH in mixed solvents cannot be accurately predicted from PL peak position under 450 nm excitation since the excited state may be shifted by solvent relaxation, thus affecting the estimation of pH as shown in Figure S16c. Therefore, the solvent polarity, together with pH, can be more accurately estimated in a slightly acidic environment by a set of spectral measurements of CD-w.

The fabrication of solid-state films is possible by embedding CDs into different matrices such as polymers [40], solid porous [41], and flexible porous matrices such as cellulose [10,13], while still retaining their optical properties such as the positions of optical transitions and high emission intensity [40]. Thus, to further develop a sensing probe based on CDs, we impregnated paper and cellulose strips with CD-w. Filter paper strips were dipped into CD-w aqueous solutions. The cellulose strips were also dipped in the CDs solutions with subsequent washing with deionized water several times to remove residual CDs at the strip surface. As can be seen in Figure S17, the concentration of initial solution slightly affected the intensity and emission color of the test strips in filter paper when varying concentration of initial CDs solutions from 1.5 to 150 μg/mL. At the same time, for cellulose strips, the concentration of impregnated CDs is independent of the concentration of the initial CDs solution. The potential for using CD@paper or a CD@cellulose probe was examined using a solution with known pH (3.0, 8.0) and checking the emission color under 265 and 365 nm UV light in comparison with a commercially available paper test strip. The photos of the samples obtained are shown in Figure S17 and S18 for the CD@paper and CD@cellulose probe, respectively.

## 4. Conclusions

In summary, we developed synthesis protocols to obtain CDs based on o-PD with variety of optical responses depending on additional precursors and reaction media changes. The obtained CDs are N-doped (N,S-doped in case of thiourea addition) spherical particles with sizes up to 15 nm with stable morphology and absorption and PL peak positions under temperature changes in a wide range (20–80 °C) and 6 month storage. The type of additional precursor (acid or thioamine) affects the morphology, including the chemical composition of the surface, which in turn governs the response to media changes. CDs synthesized from o-PD only and with addition of thiourea in water may act as an absorptive/fluorescent sensing probe for solvent polarity with $∆S/∆E_{N}^{T}\;$ of 73 and 84, respectively. Moreover, the o-PD-based CDs demonstrated both absorption and emission pH responses, wherein the emission response exhibited redistribution of PL intensity at 360 nm excitation and PL peak shift at 450 nm excitation. Based on spectral measurements, we demonstrated the possibility of dual-purpose sensing with synthesized CDs for measurement of pH in non-pure water. Moreover, these CDs can be embedded in different matrices, creating the opportunity to produce test strips that are sensitive to pH. The developed CDs are believed to serve as a dual-purpose sensing nano probe which can be implemented in various applications.

## Figures and Tables

**Table 1:** Measured and estimated values for CD-w samples in different media.

**Solvent ^a^**	$E_{N}^{T}$	${E_{N}^{T}}_{\mathrm{abs}}$	${E_{N}^{T}}_{\mathrm{PL}}$	pH (Measured)	$pH_{abs}$	$pH_{r}$	Average pH	Color ^c^
W	1.0	-	-	-	-	-	-	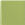
W/E	0.85 ± 0.05 ^b^	0.70 ± 0.15	1.45 ± 0.10	4.10 ± 0.05	4.4 ± 0.3	4.4 ± 0.2	4.4 ± 0.1	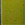
W/E	0.85 ± 0.05	0.70 ± 0.15	0.80 ± 0.10	4.70 ± 0.05	5.1 ± 0.3	4.8 ± 0.2	5.0 ± 0.1	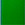
W/E	0.85 ± 0.05	0.65 ± 0.15	0.75 ± 0.10	5.90 ± 0.05	7.7 ± 0.4	6.2 ± 0.2	7.0 ± 0.1	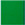
E	0.654	-	-	-	-	-	-	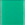

^a^ W—water, E—ethanol, W/E—mixture of water and ethanol 1 to 1; ^b^ calculated as average value between $E_{N}^{T}$ of water and ethanol weighted on their volume; ^c^ photo under 265 nm excitation.

## Supplementary Materials

The following supporting information can be downloaded at: https://www.mdpi.com/article/10.3390/nano12193314/s1. Figure S1: Scheme of o-PD oxidation and formation of carbonized domains in CDs: (a) formation of 2,3- diaminophenazine, (b) cross-linked domain, (c) linear-linked domain; Figure S2: Scheme of o-PD oxidation in the presence of thiourea and formation of carbonized N,S-doped domains in CDs. The domains can be formed both cross-linked and linear-linked 2,3-diaminophenotiazin as shown for 2,3-diaminophenazine in Figure S1; Figure S3: Size distribution from TEM images of CD-w (a), CD-HCl (b), CD-BA (d), CD-TU (d); Figure S4: AFM images and heights distributions (insets) of CD-w (a), CD-HCl (b), CD-BA (d), CD-TU (d); Figure S5: (a) Full XPS survey for CD-w (black), CD-HCl (blue), CD-BA (red), CD-TU (green), and Si substrate (light grey). (b) Chemical composition of CDs. High resolution XPS of C1s (c) and N1s (d); Figure S6: Raman spectra (excitation wavelength 514 nm) of CD samples; Figure S7: (a) PL spectra of CD-w excited at 450 nm measured at different temperatures in range from 20 to 80 °C. Temperatures are shown in legend. (b) Absorption and PL spectra of CD-w stored at different periods of time (shown in legend); Figure S8: PL excitation-emission maps of CD-w dissolved in toluene (a), chloroform (b), acetone (c), acetonitrile (d), isopropanol (e), ethanol (f); Figure S9: PL excitation-emission maps of CD-TU dissolved in toluene (a), chloroform (b), acetone (c), acetonitrile (d), isopropanol (e), ethanol (f); Figure S10: Normalized PL spectra excited at 360 nm of CD-w (a) and CD-TU (b) dissolved in different solvents; Figure S11: Photos of CD-w (a, b) and CD-TU (c, d) taken under 265 nm (a, c) and 365 nm (b, d) UV lamps excitation. Solvents are listed on photo; Figure S12: The ratio of optical density monitored at 485 and 460 nm (O.D. @485/ O.D. @460) versus pH value of the CD-w aqueous solutions; Figure S13: PL excitation emission maps of CD-w aqueous solutions with different pH: (a) 3.0, (b) 4.0, (c) 4.3, (d) 4.7, (e) 4.9, (f) 5.0, (g) 6.0, (h) 7.0, (i) 8.0; Figure S14: Absorption (a, c, e, g) and PL excited at 450 nm (b, d, f, h) of CD-w with addition of ions: Ca^2+^ (a, b), Co^2+^ (c, d), Cu^2+^ (e, f), and Fe^3+^ (g, h); Figure S15: (a) Absorption spectra, (b, c) PL spectra excited at 360 nm (b) and 450 nm (c) of CD-w dissolved in a mixture of ethanol and water with pH presented in the legend; Figure S16: The comparison of measured optical parameters of CD-w dispersed in mixture of water and ethanol with different pH: 4.1 (red), 4.7 (green), and 5.9 (blue) with dependencies obtained for: (a) absorption (red circles) and PL peak position excited at 450 nm (black squares), (b) PL intensity ratio of the peaks centered at 535 and 445 nm, (c) PL peak position while excited at 450 nm. By grey lines the optical parameters for CD-w dispersed in deionized water are shown; Figure S17: Photos of pH sensing probe based on CD-w embedded in filter paper under daylight, 265 nm UV light, and 365 nm UV light. 1 commercial paper strip, 2, 3, 4—paper strips from CD-w solutions with concentrations 150, 15, 1.5 μg/mL, respectively; Figure S18: Photos of commercial paper strip (1) and pH sensing probe based on CD-w embedded in cellulose strip under daylight (2), 265 nm UV light (3), and 365 nm UV light (4). Table S1: Amount of precursors in mg and mmol used in synthesis; Table S2: Optical parameters of CDs based on o-PD; Table S3: Optical parameters of CD-w under different storing conditions (temperature and period); Table S4: PL QY for CD-w dispersed in different solvents.
