# Supplementary material for: Dual-Purpose Sensing Nanoprobe Based on Carbon Dots from o-Phenylenediamine: pH and Solvent Polarity Measurement
Source: Nanomaterials (Basel). 2022 Sep 23;12(19):3314. doi: 10.3390/nano12193314 (PMC9565920; doi:10.3390/nano12193314)
Supplement: Supplementary file 1 [file nanomaterials-12-03314-s001.zip › nanomaterials-1920496-supplementary.pdf]

**Dual-purpose sensing nanoprobe based on carbon dots from o-phenylenediamine: pH and solvent polarity measurement**

Anna A. Vedernikova [1†], Mikhail D. Miruschenko [1†], Irina A. Arefina[1], Anton A. Babaev[1], Evgeniia A. Stepanidenko[1], Sergei A. Cherevkov[1], Igor G. Spiridonov[1], Denis V. Danilov[2], Aleksandra V. Koroleva [2], Evgeniy V. Zhizhin [2], Elena V. Ushakova[1*]

[1] International Research and Education Centre for Physics of Nanostructures, ITMO University, 197101 Saint Petersburg, Russia;

[2] Research Park, Saint Petersburg State University, 199034 Saint Petersburg, Russia;

* Correspondence: elena.ushakova@itmo.ru

† - These authors contributed equally to this work.

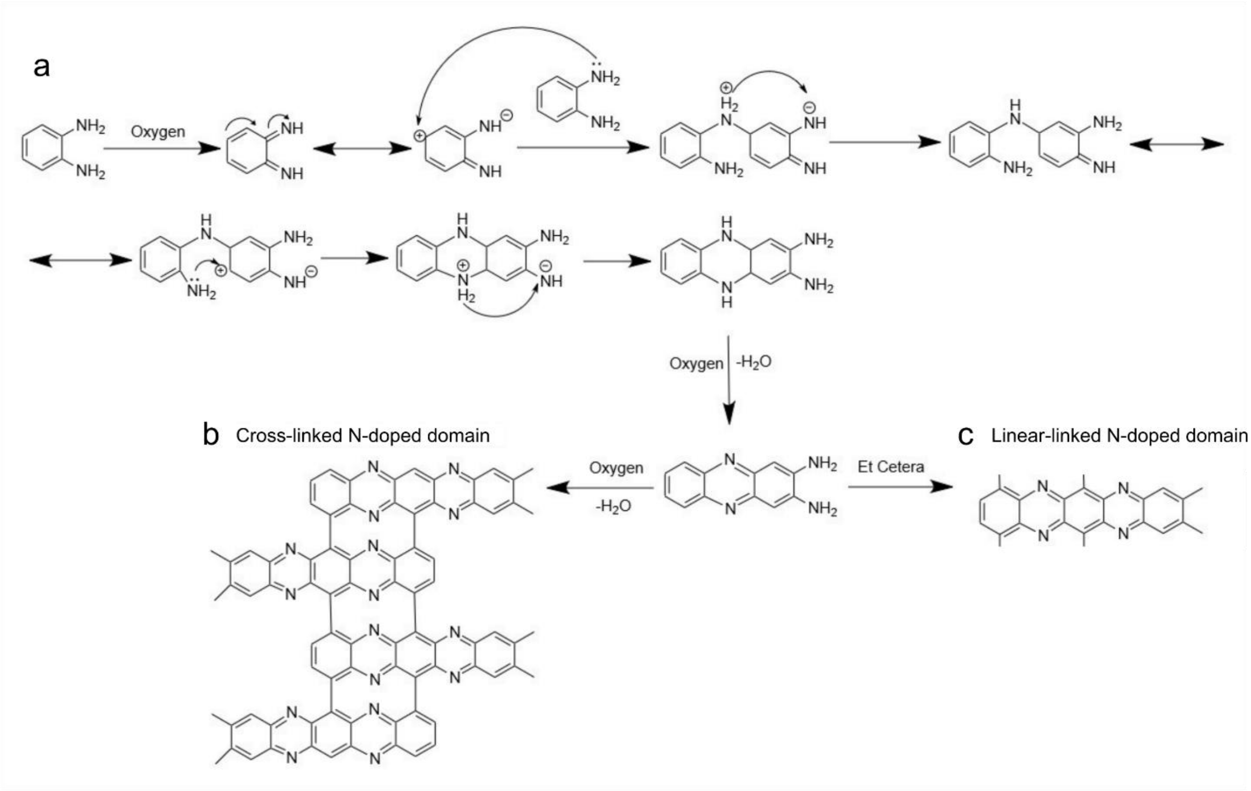


Figure S1. Scheme of o-PD oxidation and formation of carbonized domains in CDs: (a) formation of 2,3-diaminophenazine, (b) cross-linked domain, (c) linear-linked domain.

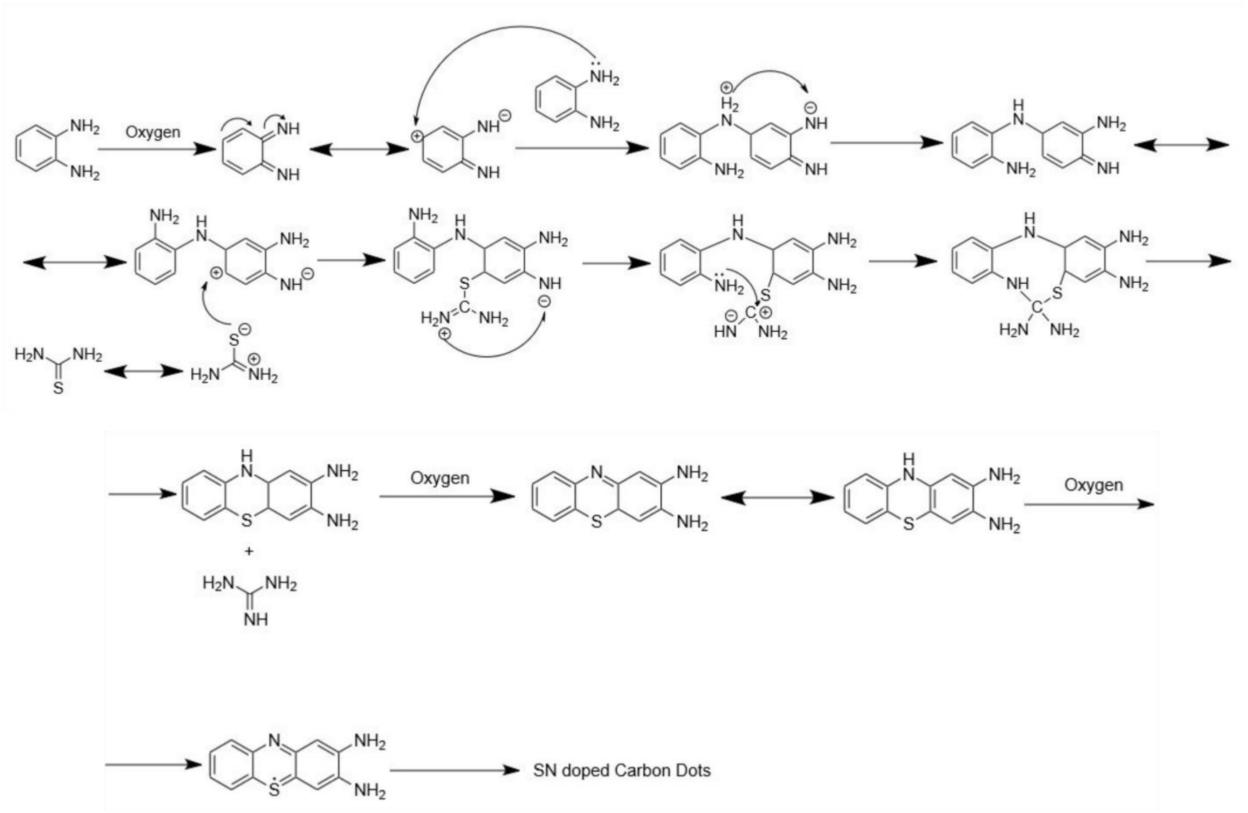


Figure S2. Scheme of o-PD oxidation in the presence of thiourea and formation of carbonized N,S-doped domains in CDs. The domains can be formed both cross-linked and linear-linked 2,3-diaminophenotiazin as shown for 2,3-diaminophenazine in Figure S1.

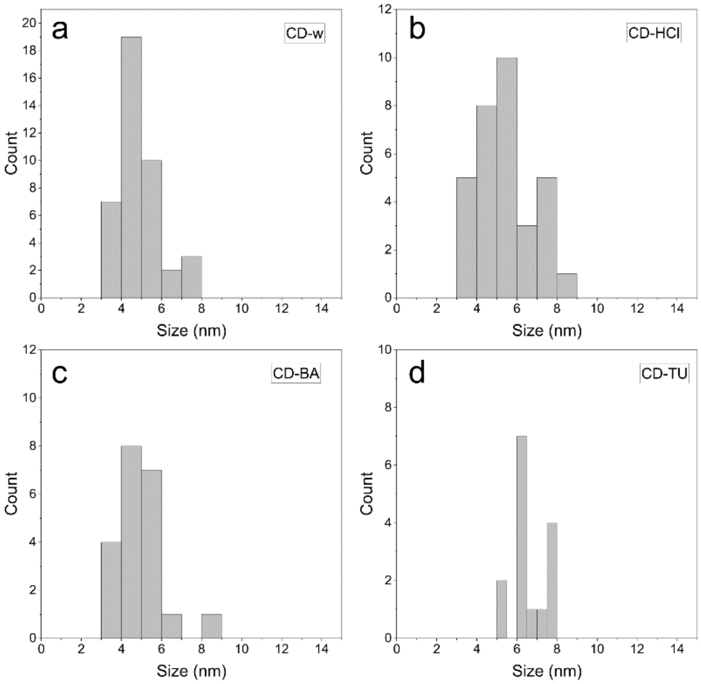


Figure S3. Size distribution from TEM images of CD-w (a), CD-HCl (b), CD-BA (d), CD-TU (d)

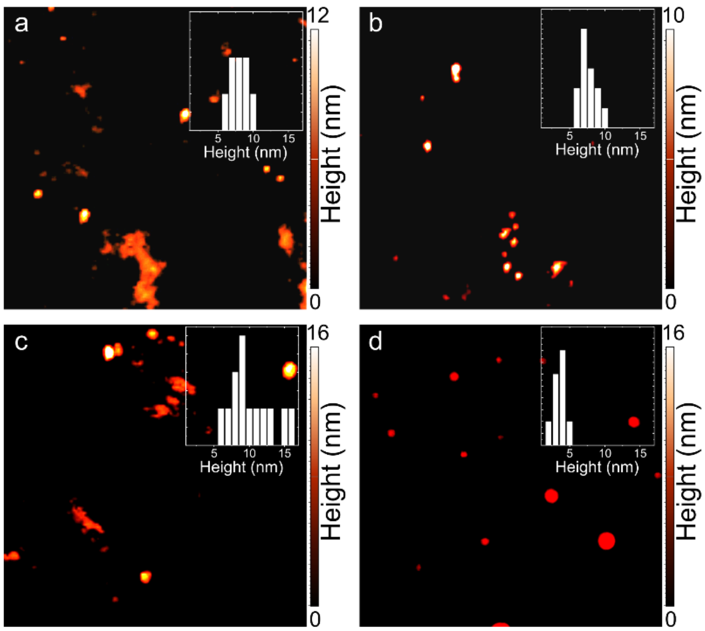


Figure S4. AFM images and heights distributions (insets) of CD-w (a), CD-HCl (b), CD-BA (d), CD-TU (d)

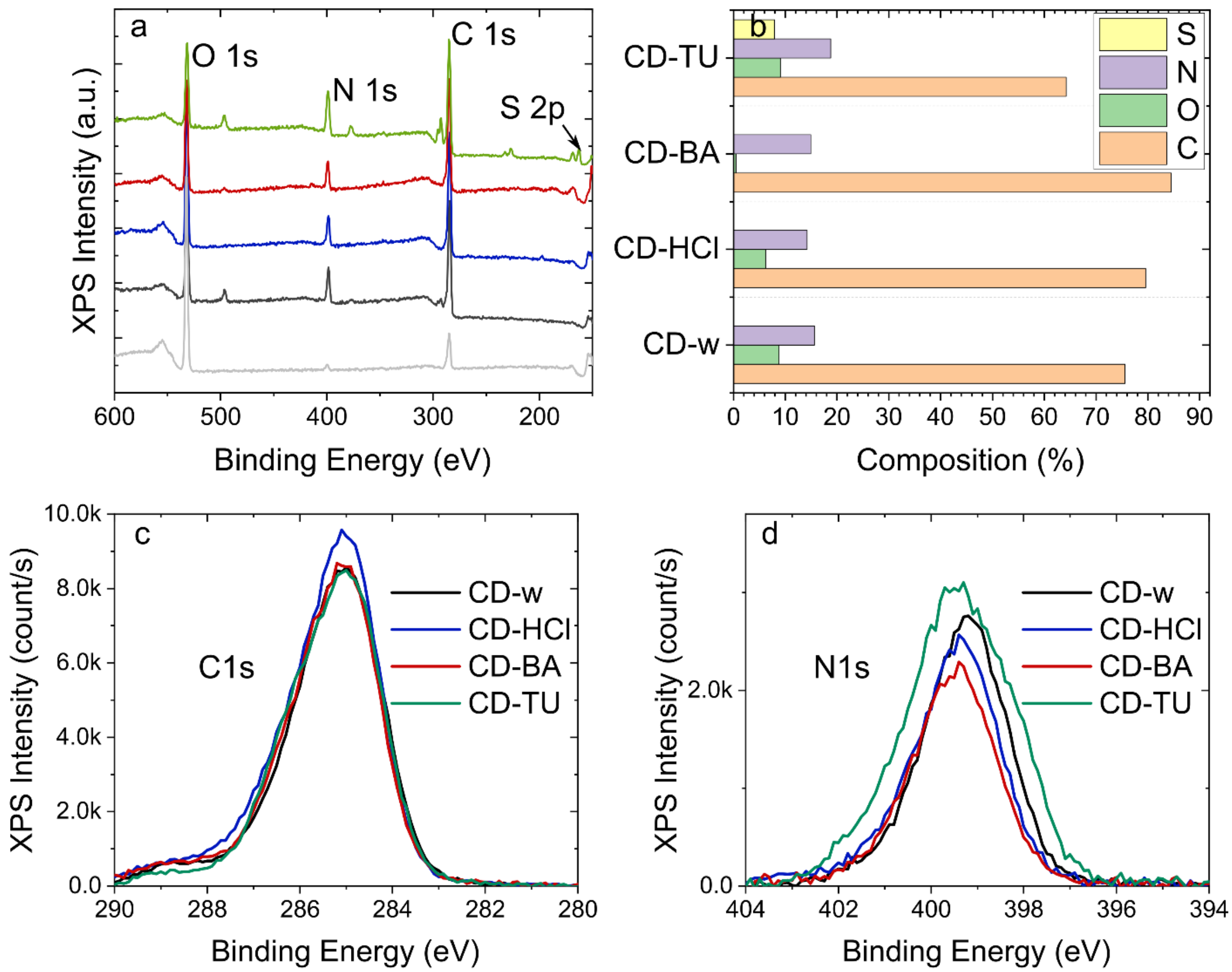


Figure S5. (a) Full XPS survey for CD-w (black), CD-HCl (blue), CD-BA (red), CD-TU (green), and Si substrate (light grey). (b) Chemical composition of CDs. High resolution XPS of C1s (c) and N1s (d).

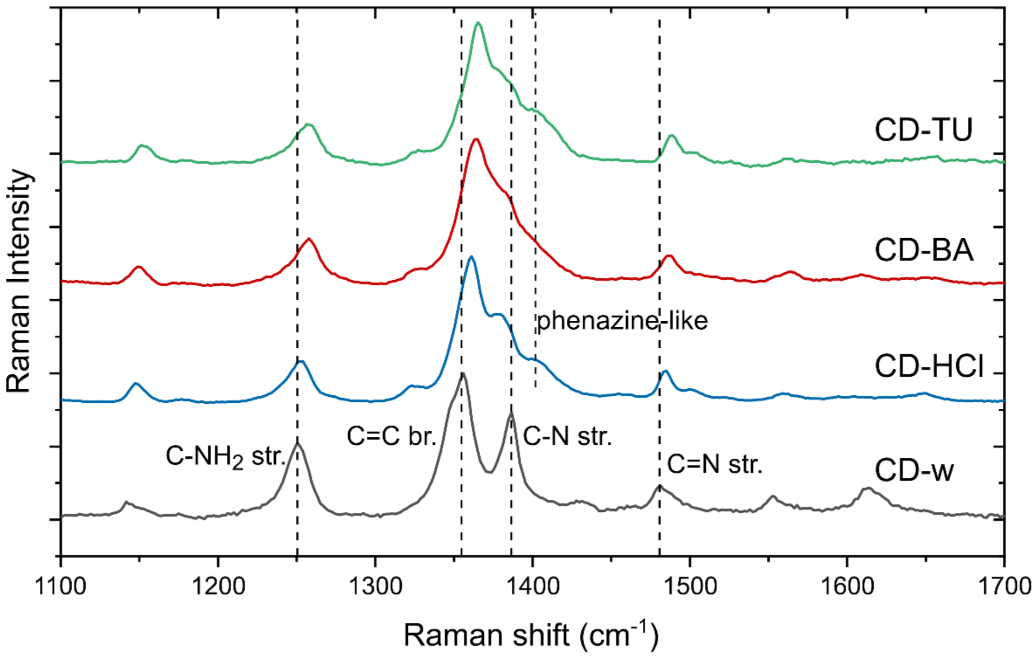


Figure S6. Raman spectra (excitation wavelength 514 nm) of CD samples.

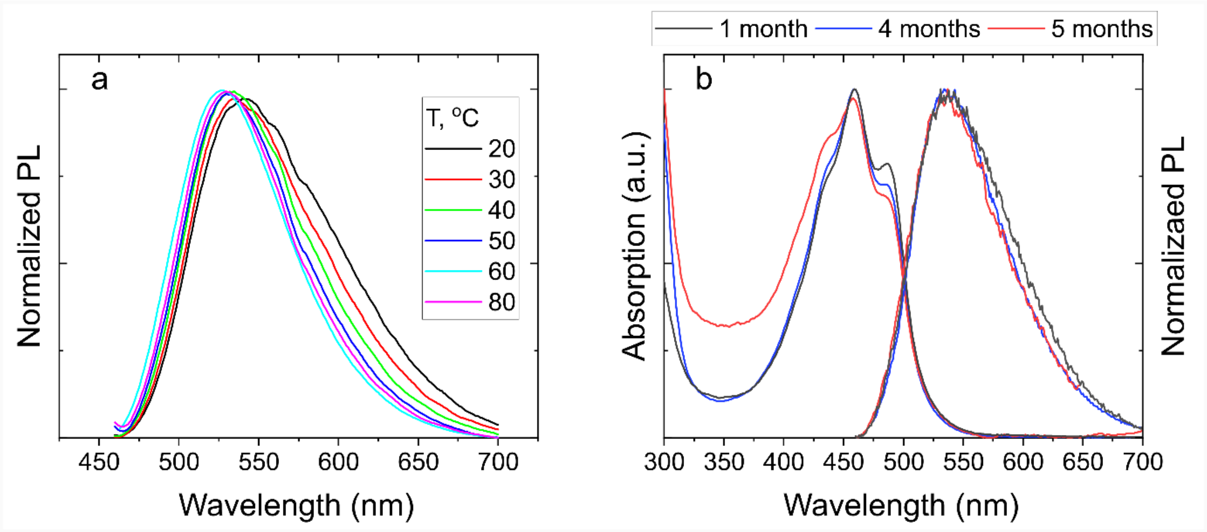


Figure S7. (a) PL spectra of CD-w excited at 450 nm measured at different temperatures in range from 20 to 80 °C. Temperatures are shown in legend. (b) Absorption and PL spectra of CD-w stored at different periods of time (shown in legend).

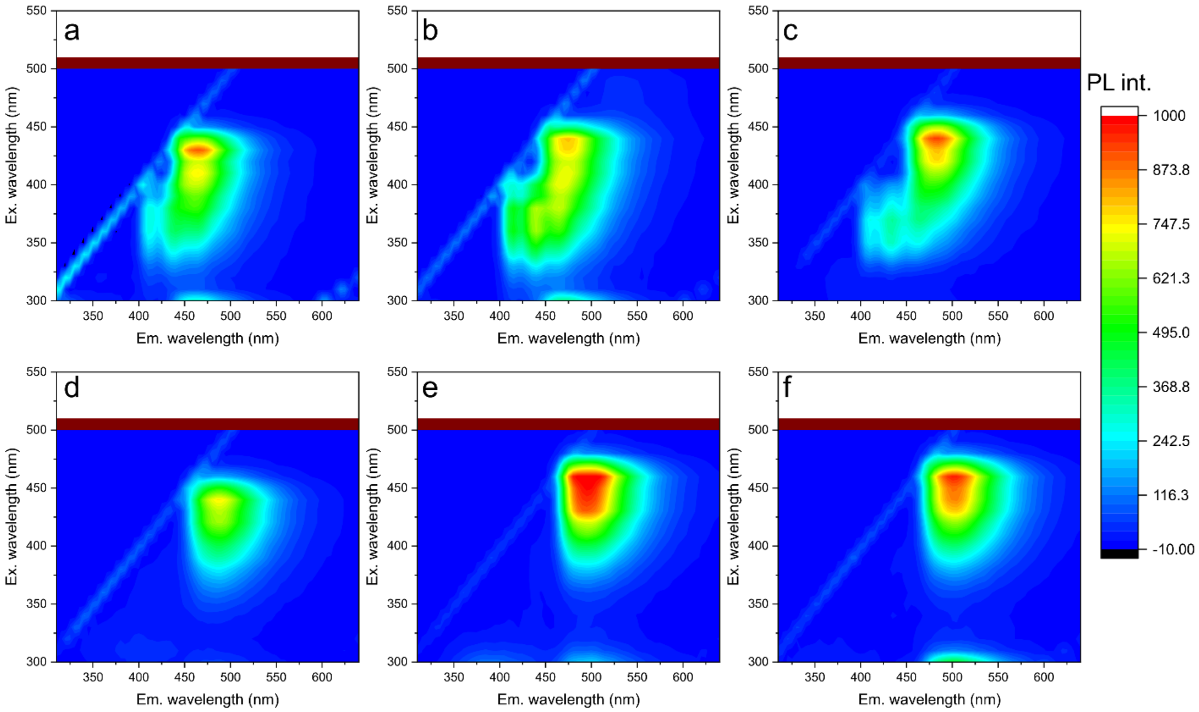


Figure S8. PL excitation-emission maps of CD-w dissolved in toluene (a), chloroform (b), acetone (c), acetonitrile (d), isopropanol (e), ethanol (f).

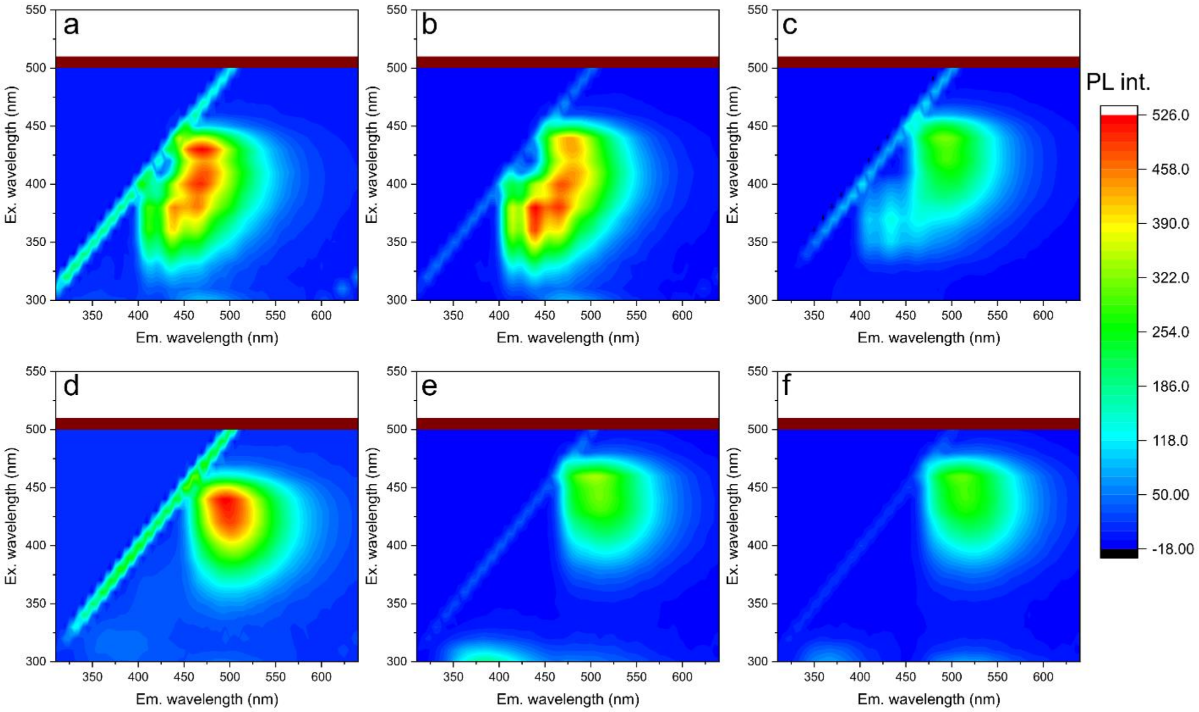


Figure S9. PL excitation-emission maps of CD-TU dissolved in toluene (a), chloroform (b), acetone (c), acetonitrile (d), isopropanol (e), ethanol (f).

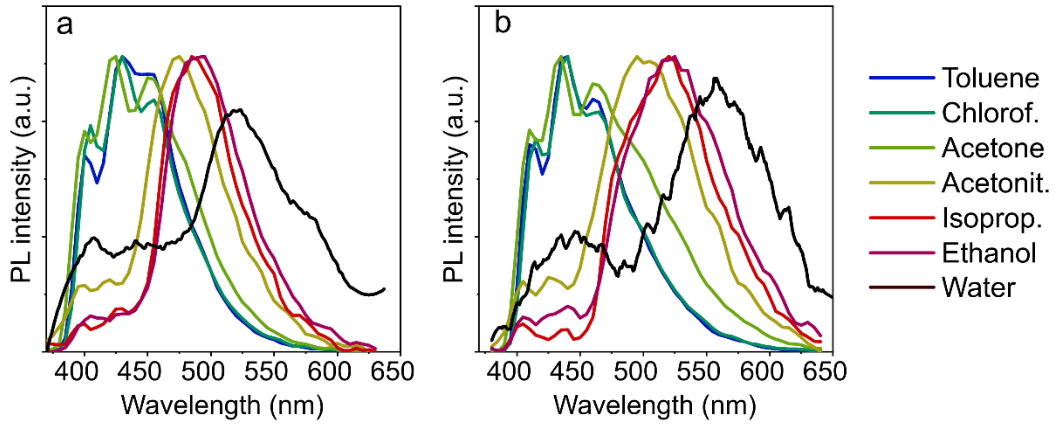


Figure S10. Normalized PL spectra excited at 360 nm of CD-w (a) and CD-TU (b) dissolved in different solvents.

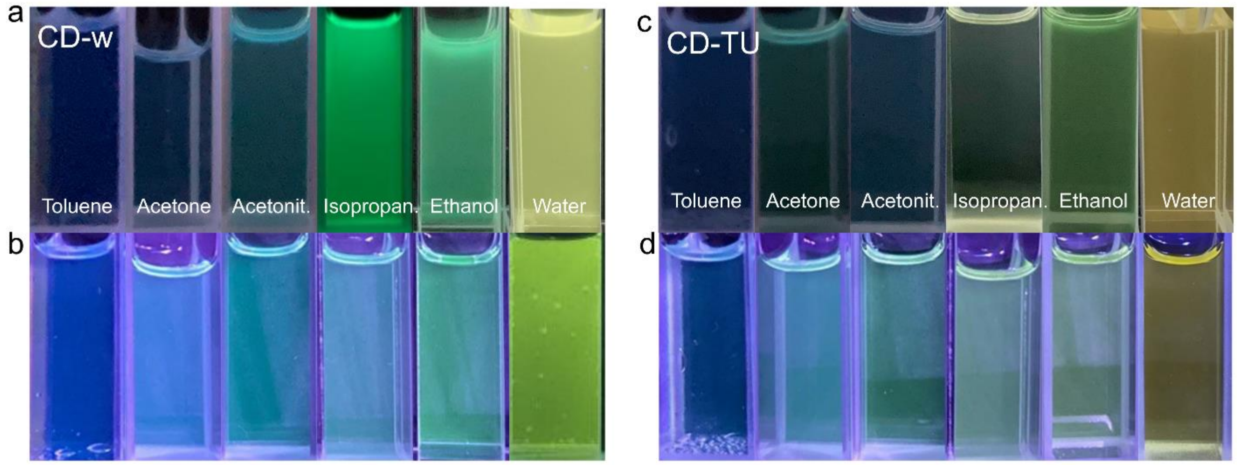


Figure S11. Photos of CD-w (a, b) and CD-TU (c, d) taken under 265 nm (a, c) and 365 nm (b, d) UV lamps excitation. Solvents are listed on photo.

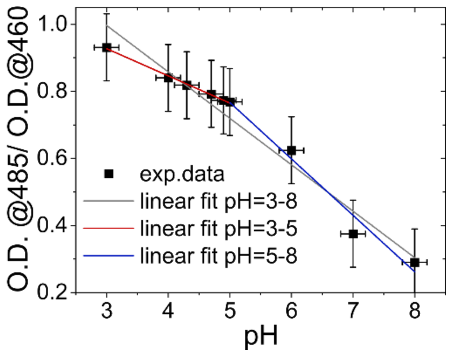


Figure S12. The ratio of optical density monitored at 485 and 460 nm (O.D. @485/ O.D. @460) versus pH value of the CD-w aqueous solutions. Experimental data was fitted by linear functions: for pH = 3-8 $O.D._{485}/O.D._{460} = 1.40 - 0.14 \cdot pH$, for pH < 5.0 $O.D._{485}/O.D._{460} = 1.17 - 0.08 \cdot pH$, and for base pH > 5.0 $O.D._{485}/O.D._{460} = 1.61 - 0.17 \cdot pH$

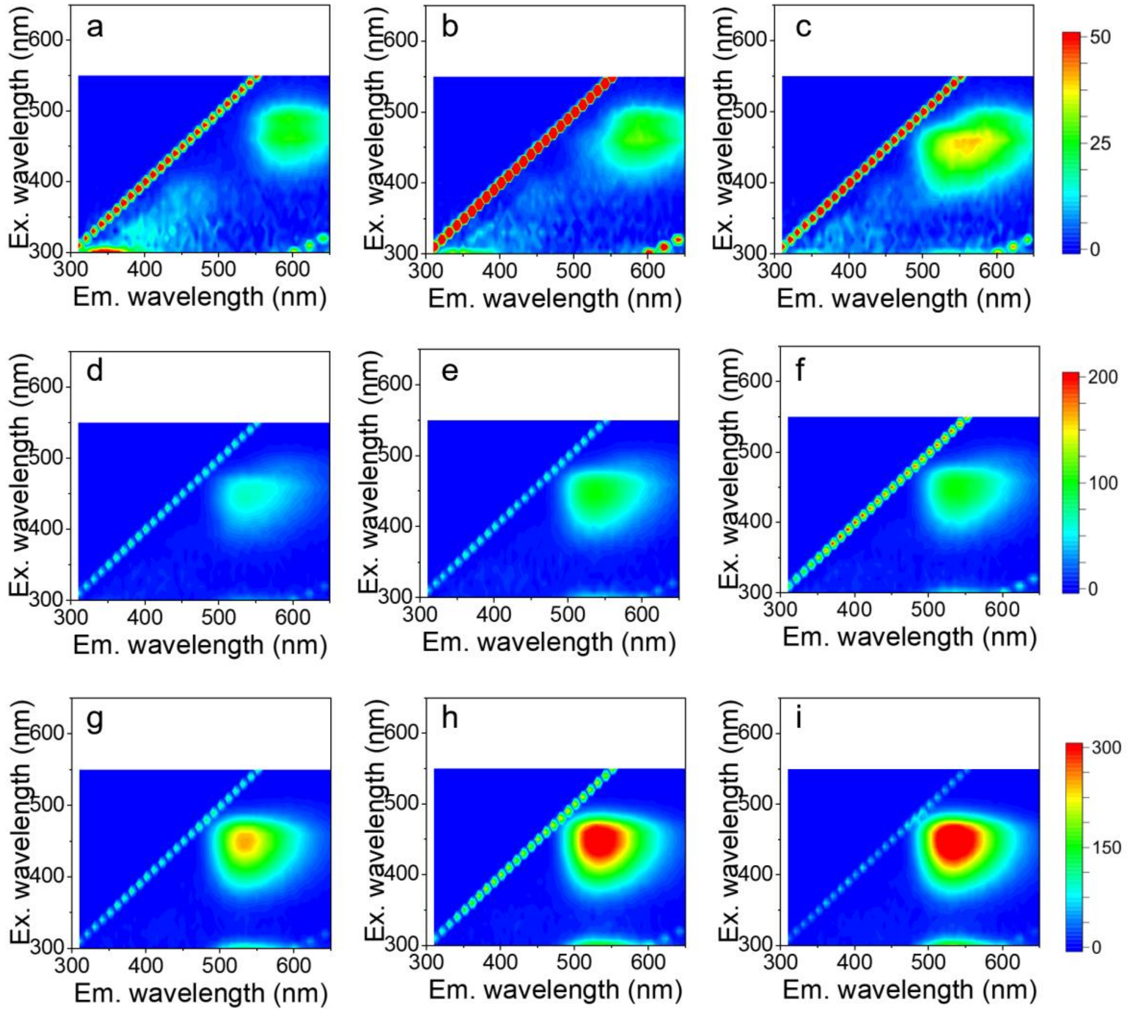


Figure S13. PL excitation emission maps of CD-w aqueous solutions with different pH: (a) 3.0, (b) 4.0, (c) 4.3, (d) 4.7, (e) 4.9, (f) 5.0, (g) 6.0, (h) 7.0, (i) 8.0.

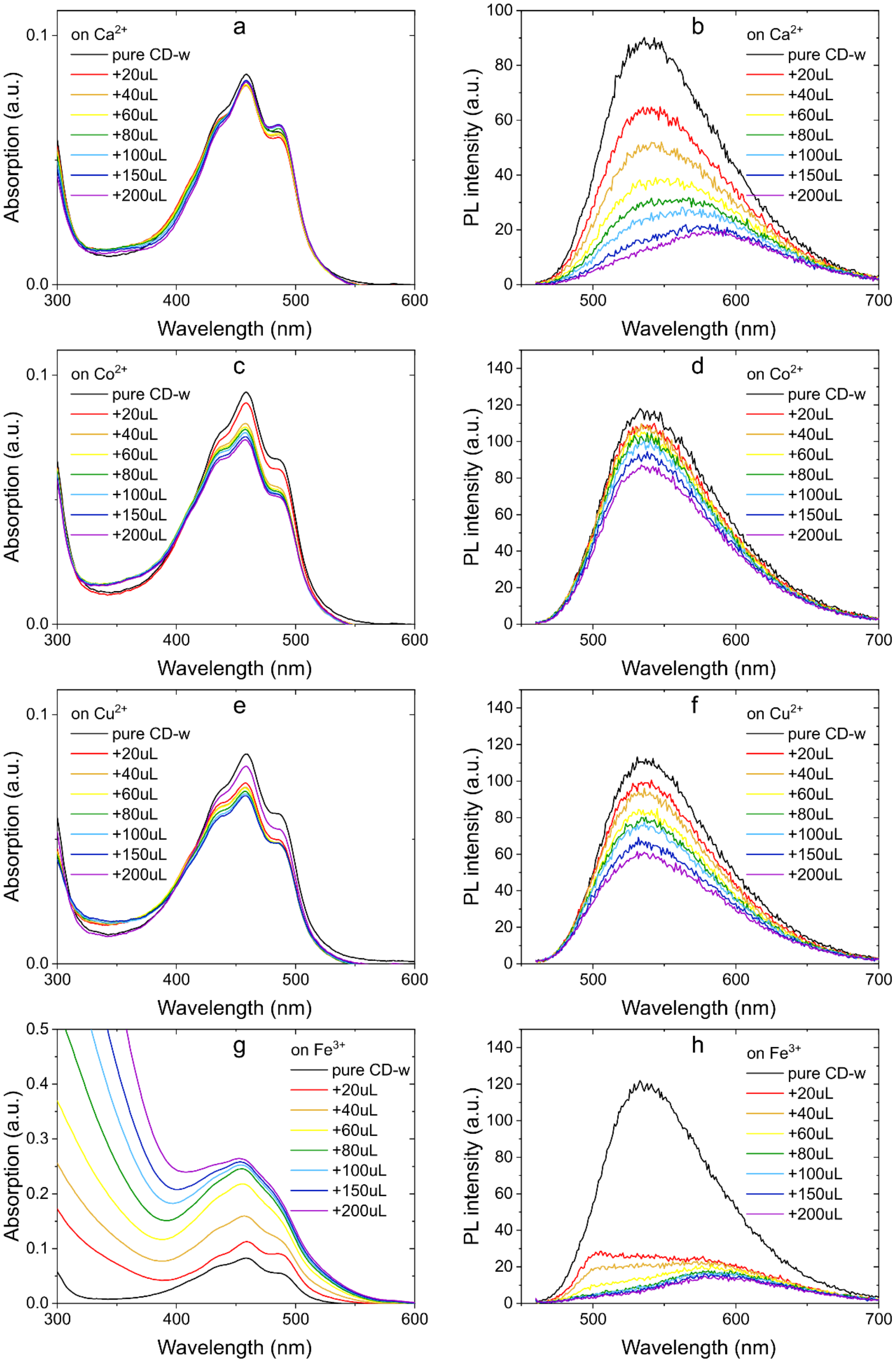


Figure S14. Absorption (a, c, e, g) and PL excited at 450 nm (b, d, f, h) of CD-w with addition of ions: $Ca^{2+}$ (a, b), $Co^{2+}$ (c, d), $Cu^{2+}$ (e, f), and $Fe^{3+}$ (g, h). To CD-w solution in water at pH=5.1 a different amount of 0.01 M solutions of salts ($CuSO_4$, $FeCl_3$, $CaCl_2$, $CoCl_2$) has been added.

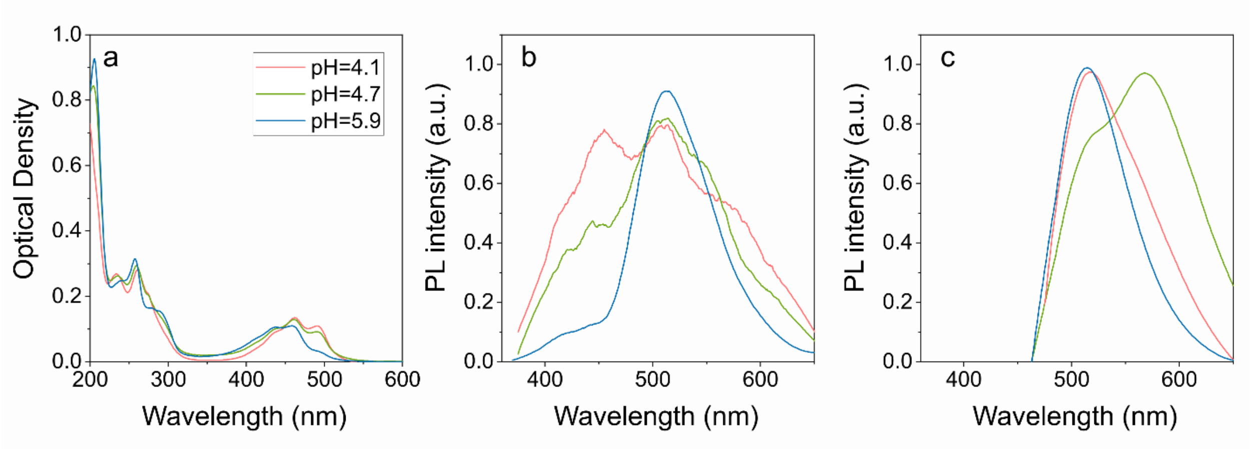


Figure S15. (a) Absorption spectra, (b, c) PL spectra excited at 360 nm (b) and 450 nm (c) of CD-w dissolved in a mixture of ethanol and water with pH presented in the legend.

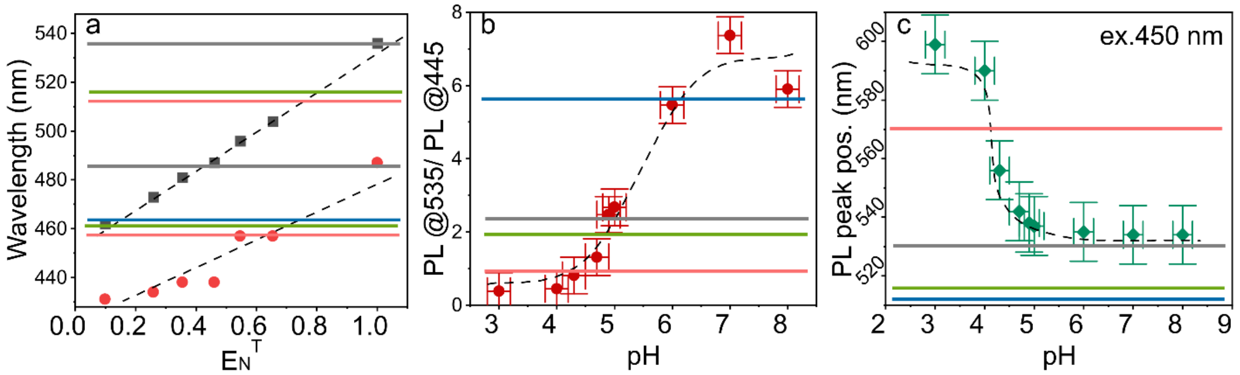


Figure S16. The comparison of measured optical parameters of CD-w dispersed in mixture of water and ethanol with different pH: 4.1 (red), 4.7 (green), and 5.9 (blue) with dependencies obtained for: (a) absorption (red circles) and PL peak position excited at 450 nm (black squares), (b) PL intensity ratio of the peaks centered at 535 and 445 nm, (c) PL peak position while excited at 450 nm. By grey lines the optical parameters for CD-w dispersed in deionized water are shown.

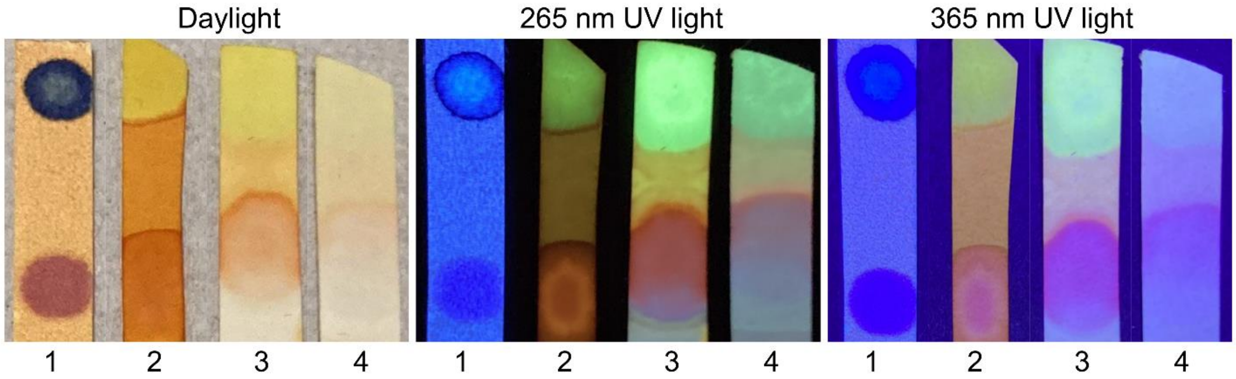


Figure S17. Photos of pH sensing probe based on CD-w embedded in filter paper under daylight, 265 nm UV light, and 365 nm UV light. 1-commercial paper strip, 2, 3, 4 – paper strips from CD-w solutions with concentrations 150, 15, 1.5 μg/mL, respectively.

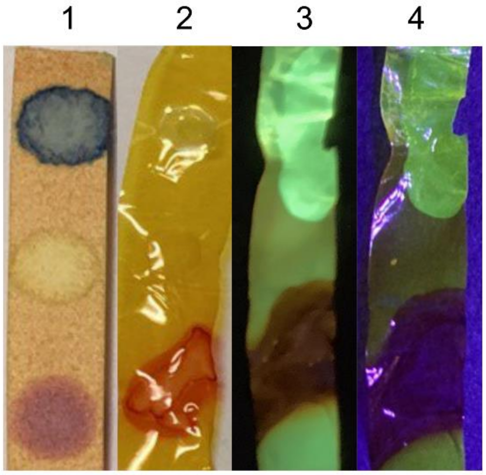


Figure S18. Photos of commercial paper strip (1) and pH sensing probe based on CD-w embedded in cellulose strip under daylight (2), 265 nm UV light (3), and 365 nm UV light (4).

Table S1. Amount of precursors in mg and mmol used in synthesis

| | CD-w | | CD-HCl | | CD-BA-w | | CD-TU-w | |
|---|---|---|---|---|---|---|---|---|
| | mg | mmol | mg | mmol | mg | mmol | mg | mmol |
| o-PD | 500 | 4.624 | 500 | 4.624 | 500 | 4.624 | 500 | 4.624 |
| benzoic acid | | | | | 560 | 4.586 | | |
| HCl pH=2 | | | | 0.250 | | | | |
| Thiourea | | | | | | | 700 | 9.196 |
| Atom of oxygen* | | 0.536 | | 0.536 | | 0.536 | | 0.536 |
| Solvent | 25 mL | | 25 mL | | 25 mL | | 25 mL | |

* The amount of oxygen was calculated from residual volume of autoclave of 25 mL and amount of oxygen present in water at ambient conditions.

Table S2. Optical parameters of CDs based on o-PD

| Sample name | Abs peak position UV, nm | Abs peak position Vis, nm | Abs peak position (additional*), nm | PL peak position ex.280 nm, nm | PL peak position ex.450 nm, nm | PL lifetime, ns | PL QY ex.450 nm, % |
|---|---|---|---|---|---|---|---|
| CD-w | 260 | 435, 460, 485 | - | 535 | 535 | 2.3 ± 0.3 | 5.4 |
| CD-HCl | 260 | 435, 460, 485 | - | 535 | 535 | 2.2 ± 0.3 | 5.3 |
| CD-BA | 280 | 435, 460, 485 | - | 360 | 570 | 1.7 ± 0.3 | 4.5 |
| CD-TU | 290 | 440 | 350-380 | 550 | 550 | 1.8 ± 0.3 | 5.0 |

Table S3. Optical parameters of CD-w under different storing conditions (temperature and period)

| | t=20 °C | t=30 °C | t=40 °C | t=50 °C | t=60 °C | t=80 °C | 1 month | 4 months | 5 months |
|---|---|---|---|---|---|---|---|---|---|
| Abs peak, nm | 460 | 460 | 459 | 458 | 456 | 455 | 460 | 460 | 458 |
| PL peak ex.450 nm, nm | 537 | 535 | 533 | 531 | 527 | 530 | 535 | 535 | 535 |

Table S4. PL QY for CD-w dispersed in different solvents

| Solvent | Toluene | Chloroform | Acetone | Acetonitrile | Isopropanol | Ethanol | Water |
|---|---|---|---|---|---|---|---|
| $E_N^T$ | 0.099 | 0.259 | 0.355 | 0.46 | 0.546 | 0.654 | 1.0 |
| PL QY ex.450 nm, % | 14.0 | 10.2 | 16.4 | 16.5 | 21.5 | 17.5 | 5.4 |
